# Trends in Graves’ orbitopathy research in the past two decades: a bibliometric analysis

**DOI:** 10.5935/0004-2749.20220081

**Published:** 2025-02-11

**Authors:** Khaled A. Elubous, Ali D. Alebous, Hebah A. Abous, Rawan A. Elubous, Lana A. Alebous, Taher O. Alshammari

**Affiliations:** 1 Department of Ophthalmology, University of Jordan, Amman, Jordan; 2 Department of Surgery, King Hussein Cancer Center, Amman, Jordan; 3 Department of Dermatology, Jordanian Royal Medical Services, Amman, Jordan; 4 Department of Radiology, University of Jordan, Amman, Jordan; 5 School of Medicine, University of Jordan, Amman, Jordan

**Keywords:** Graves ophthalmopathy, Bibliometrics, Oftalmopatia de Graves, Research, Oftalmopatia de Graves, Bibliometria, Oftalmopatia de Graves, Pesquisa

## Abstract

**Purpose:**

This study was conducted to identify trends in Graves’ orbitopathy research in the past two decades and to elaborate on hot topics in the field.

**Methods:**

The Web of Science database was used to extract articles on Graves’ orbitopathy or its synonyms. Full data and references were exported to VOSviewer software to be analyzed. Visualization maps and charts were constructed accordingly.

**Results:**

We retrieved 1067 articles on Graves’ orbitopathy from the Web of Science database. The United States ranked first in terms of the article count (25), followed by Italy (141) and the People’s Republic of China (120). Wiersinga’s and the University of Amsterdam’s articles received the highest citation count (1509 and 3052, respectively). The University of Pisa and *Thyroid* published the highest number of articles (65 and 93, respectively). Co-authorship analysis showed four clusters of country collaborations: red cluster, European countries; green cluster, the United States, Brazil, Canada, South Korea, and Taiwan; a yellow cluster, People’s Republic of China; and blue cluster, Japan, Australia, and Poland. Keyword analysis revealed five clusters of topics: pathogenesis, management, association, quality of life, and surgery. Analysis of co-cited references also revealed five clusters: pathogenesis, management, risk factors, clinical assessment, and surgical management.

**Conclusion:**

Research on Graves’ orbitopathy has grown during the past two decades. Hot research topics are pathogenesis, management, risk factors, quality of life, and complications. Research trends have changed in the past two decades. Increasing interest in exploring Graves’ orbitopathy mechanisms and associations is evident. European countries are cooperating in this field of research. The United States has established more extensive international cooperation than other countries. We believe that more international collaboration involving developing countries is required.

## Abstract (pt)

**Objetivo:**

Identificar tendências no campo de pesquisa da orbitopatia de Graves nas últimas duas décadas e analisar os ramos de maior concentração de pesquisas nessa área.

**Métodos:**

O banco de dados Web of Science foi usado para extrair artigos com “orbitopatia de Graves” ou seus sinônimos no título. Dados completos e referências foram exportados para o programa VOSviewer para serem analisados. Mapas e gráficos de visualização foram construídos a partir desses dados.

**Resultados:**

Foram obtidos 1067 artigos sobre a orbitopatia de Graves a partir do banco de dados Web of Science. Os EUA ficaram em primeiro lugar em termos de número de publicações, seguidos pela Itália e pela República Popular da China. Dentre os autores, os artigos de Wiersinga WM tiveram o maior número de citações. Quanto às instituições, os artigos da Universidade de Amsterdã tiveram o maior número de citações, mas a Universidade de Pisa publicou o maior número de artigos. Dentre os periódicos, a revista *Thyroid* publicou o maior número de artigos. A análise de coautoria mostrou quatro agrupamentos de colaboração entre países. O primeiro agrupamento engloba países europeus; o segundo engloba os EUA, Brasil, Canadá, Coreia do Sul e Taiwan. A República Popular da China compreende um agrupamento por si só. O quarto agrupamento inclui Japão, Austrália e Polônia. A análise das palavras-chave revelou cinco agrupamentos de tópicos de palavras-chave: patogênese, gerenciamento, associação, qualidade de vida e cirurgia. A análise das referências citadas em conjunto revelou cinco agrupamentos: patogênese, manejo, fatores de risco, avaliação clínica e manejo cirúrgico.

**Conclusão:**

A pesquisa no campo da orbitopatia de Graves cresceu nos últimos vinte anos. Os tópicos com a maior concentração de pesquisas são: patogênese, gerenciamento, fatores de risco, qualidade de vida e complicações. As tendências de pesquisa mudaram nas últimas duas décadas. Ficou evidente um aumento do interesse em explorar os mecanismos e associações da orbitopatia de Graves. Observou-se uma cooperação entre países europeus neste campo de pesquisa. Os EUA estabeleceram uma cooperação internacional mais ampla que outros países. Acreditamos que mais colaboração internacional envolvendo países em desenvolvimento seria recomendável.

## INTRODUCTION

Graves’ orbitopathy, also called thyroid eye disease (TED), is an autoimmune disease that mostly affects patients with thyrotoxicosis or a previous history of hyperthyroidism^(1)^. The exact underlying molecular mechanism is unclear. However, complex interactions occur between orbital fibroblasts, T-cells, thyrotropin receptors, and cytokines. Studies have shown a marked decline in TED prevalence between 1960 and 1990 in Western European countries, attributed to several factors, such as improvement of diagnostic tools and management of dysthyroid and TED as well as a reduction in the smoking rate^(2)^. Major risk factors for the development of TED are tobacco smoking, advanced age of onset, female gender, and radioiodine therapy^(3)^. The main treatment includes corticosteroids, radiotherapy, and surgical therapy. An advanced understanding of the underlying molecular mechanism and risk factors of TED has obvious implications on management patterns^(4)^. Understanding the molecular mechanism of TED has greatly improved in recent years^(5)^. New immune modulator drugs have emerged, such as tepratomumab, selenium, and rituximab.

Bibliometric analysis gives a broader understanding of the research trends and hot research areas in a particular topic. This study identified research trends in TED in the past two decades. We also investigated the collaboration between countries and identified leading journals, institutes, and authors in this field. The top-cited articles and co-cited references were also identified.

## METHODS

The search for articles on TED, published between 2000 and 2019, was performed on May 12, 2020, using the Web of Science (WOS) database-Core Collection (Science Citation Index Expanded). All articles whose titles contained the following terms were included in our analysis: dysthyroid ophthalmopathy, thyroid-associated ophthalmopathy, Graves’ orbitopathy, orbitopathy, Graves, Graves’ ophthalmopathy, Graves’ eye, thyroid eye, thyroid-related ophthalmopathy, thyroid-related orbitopathy, and thyroid-related eye disease. No language was excluded. Other types of papers, such as editorials, reviews, and abstracts, were excluded from our analysis.

The search was conducted separately by the co-first authors, KE and AA, to double-check the results and the repeatability of the methods. An indiscriminate manual check to exclude irrelevant articles was performed. No articles were excluded after the manual check.

Results were analyzed using the WOS engine, which provided the counts of articles per year, per country, and per institution. The exportation and analysis of full records and cited references were performed using VOSviewer software version 1.6.15. Charts, tables, and knowledge visualization maps were constructed accordingly. Next, the topics of co-cited references were identified. To investigate the international collaborations between countries, we performed co-authorship analysis and co-occurrence ranking of 20 countries, each of which contributed to at least 20 articles, using the fractional counting method, rather than full counting method, of VOSviewer software (we believe fractional counting is more adequate for ranking^(6)^).

No ethical approval was needed for this study, because we used a public database (WOS) and did not involve human subjects.

## RESULTS

**Figure 1:**
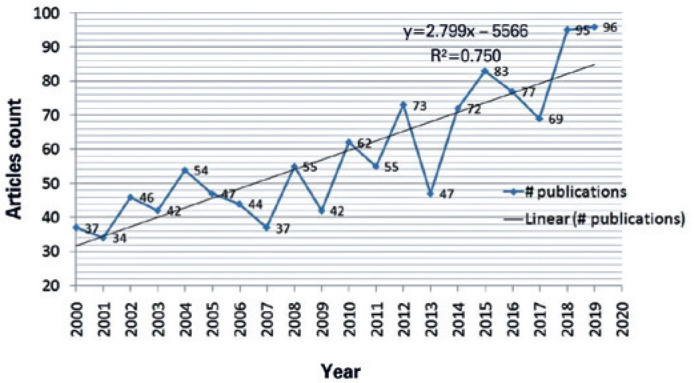
Article count per year in TED research. The highest number of published articles was in 2019 (96 articles). The number of published articles per year increased with time.

The WOS database search revealed 1067 articles with an h-index of 66. The sum of the citations was 22,060, and the average citations per article were 18.9. English was the dominant language (96.4%) used in most articles, followed by German (2%). The year 2019 had the highest number of articles (96), followed by 2018 (95). The publication count per year is represented in the chart in figure 1, and results showed that the number of articles published per year is generally increasing with time.

*y*=2.799*x* - 5566. *R*^^2^^=0.750. TED= thyroid eye disease.

### Article production and collaboration by country of origin

The United States had the highest publication count (250 articles), followed by Italy (141) and the People’s Republic of China (PRC; 120). The United States also ranked first in terms of the citation count (5833 citations), followed by Italy (4852) and the Netherlands (4120).

**Figure 2:**
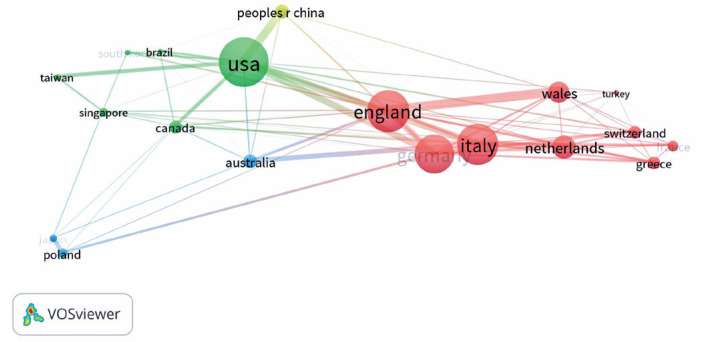
Network visualization map for the country’s collaboration in TED research. Larger nodes indicate a higher total link strength. A wider link indicates a stronger link between two countries. Each cluster of co-authorship is coded by a color. Four clusters of countries in co-authorship are coded by green, red, yellow, and blue, respectively.

As shown in figure 2, countries with higher total link strength appeared with larger nodes, while the larger width of a link indicated strong cooperation between two countries. The United States had the highest total link strength, followed by England and Italy. Co-authorship analysis showed four clusters of country collaborations designated by a distinct color: red cluster, European countries; blue cluster, the United States, Brazil, Canada, South Korea, and Taiwan; yellow cluster, PRC; and blue cluster, Japan, Australia, and Poland. Most of the research collaboration in this field occurred between developed countries.

TED= thyroid eye disease.

### Top institutes, authors, and journals for TED

The top 10 authors in this field are shown in table 1. Marcocci ranked first in terms of the article count (56 articles, 1089 citations). Wiersinga ranked first in terms of the citation count (46 articles, 1509 citations).

**Table 1:** The top 10 authors and institutes in the research field of TED

Rank	Institute	Count (%)	Country	Author	Document count (%)	Country	h-Index
1	University of Pisa	65 (5.6)	Italy	Marcocci C	56 (4.8)	Italy	60
2	University Amsterdam	53 (4.5)	Netherlands	Wiersinga WM	46 (3.9)	Netherlands	20
3	Yonsei University	44 (3.8)	South Korea	Marino M	45 (3.9)	Italy	26
4	University Calif Los Angeles	34 (2.9)	USA	Yoon JS	41 (3.5)	South Korea	20
5	Mayo Cline	32 (2.7)	USA	Eckstein A	34 (2.9)	Germany	24
6	University Insubria	29 (2.5)	Italy	Nardi M	34 (2.9)	Italy	29
7	University Michigan	25 (2.1)	USA	Kahaly G1	32 (2.7)	Germany	24
8	University Milan	24 (2.1)	Italy	Smith TJ	31 (2.7)	USA	52
9	Cardiff University	22 (1.9)	Wales	Bartalena L	30 (2.6)	Italy	51
10	Moorefield’s Eye Hosp	21 (4.5)	England	Mounts MP	29 (2.5)	Netherlands	19

TED= thyroid eye disease.

The top 10 institutes that contributed to one-third of all articles (349 of 1067) are also listed in table 1. The University of Pisa contributed to the highest number of articles (65). The University of Amsterdam had the highest citation count (3052).

Of all articles on TED, 20% were published in three journals. The number of journals that published articles on TED was 263, of which 10 published 40% of the all articles. These 10 leading journals and their impact factor, total number of citations, and articles are listed in table 2. Journals that published the highest number of articles were *Thyroid* (93), *Ophthalmic Plastic and Reconstructive Surgery* (83), and *Journal of Clinical Endocrinology Metabolism* (68). The journals with the highest citation count were *Journal of Clinical Endocrinology Metabolism* (3952)*, Thyroid* (2613), and *Clinical Endocrinology* (1233).

**Table 2:** Top 10 journals in TED with their document count, citation count, and impact factor

Title	Articles	Citations		IF 2019		IF
Thyroid	**93**	2613		5.227		6.29
Ophthalmic Plastic and Reconstructive Surgery	83	936			^*^	
Journal of Clinical Endocrinology Metabolism	68	**3952**		5.399		5.879
Eye	41	583		2.455		2.732
Investigative Ophthalmology Visual Science	38	750		3.47		3.659
Journal of Endocrinological Investigation	37	650		3.397		3.016
Clinical Endocrinology	34	1233		3.38		3.366
Ophthalmology	33	1132		8.47		8.339
Graefes Archive for Clinical and Experimental Ophthalmology	33	825		2.396		2.258
British Journal of Ophthalmology	27	974		3.611		3.402

Data are from the 2019 edition of *Journal Citation Reports*.

IF 2019= impact factor 2019; IF= 5-year impact factor.

* = data from the 2002 edition of *Journal Citation Reports*; IF= 0.588. TED= thyroid eye disease.

### Keyword analysis

**Figure 3:**
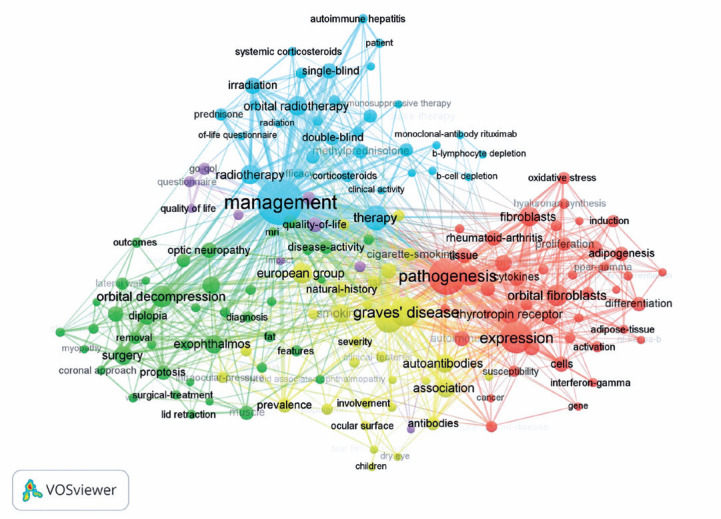
Network visualization map of highly co-occurring keywords in TED research. Keywords associated together formed a cluster. Five clusters of keywords were formed, and each cluster was coded by a color. Nodes with a larger size indicate a higher occurrence of the keyword. An increase in the width of a link indicates a higher total link strength.

Of the 2650 keywords used in all articles, 174 occurred 10 times or more. Analysis of these keywords created five clusters, named according to the common relationship of words between them: red cluster, pathogenesis; blue cluster, management; green cluster, surgery; yellow cluster, associations; and purple cluster, quality of life. A network visualization map of highly cooccurring keywords in TED research is shown in figure 3. Analysis of the overlay visualization map illustrated the average publication per year frame for keywords. For example, the keyword average publication year of 2014 was coded in red, while the keyword average publication year of 2008 was coded in blue. Dark-blue-coded keywords included corticosteroids, irradiation, coronal approach, INF-γ, PPAR-γ, cigarette smoking, wall, and muscle. Red-coded keywords included mechanism, oxidative stress, inflammation, orbital fibroblasts, NFkappa B, proliferation, risk factors, rituximab, European group, prediction, impact, dry eye, ocular surface, tear film osmolarity, autoimmune hepatitis, and efficacy.

TED= thyroid eye disease.

**Figure 4:**
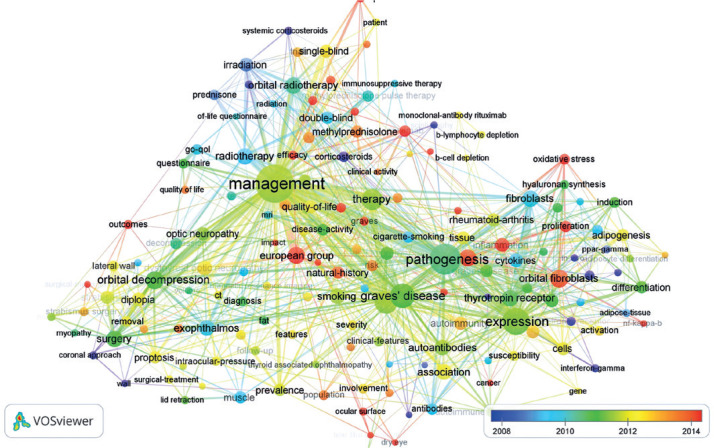
Overlay visualization map of highly co-occurring keywords in TED research. Red nodes indicate that the average publication year is 2014, while dark-blue nodes indicate the average publication year is 2009. The size of the node represents a positive relationship with the occurrence count. A larger node indicates higher occurrence times.

An overlay visualization map of highly co-occurring keywords in TED research is shown in figure 4. Red nodes indicate that the average publication year is 2014, while dark-blue nodes indicate the average publication year is 2009.

TED= thyroid eye disease.

### Highly cited articles and co-citation analysis

**Figure 5:**
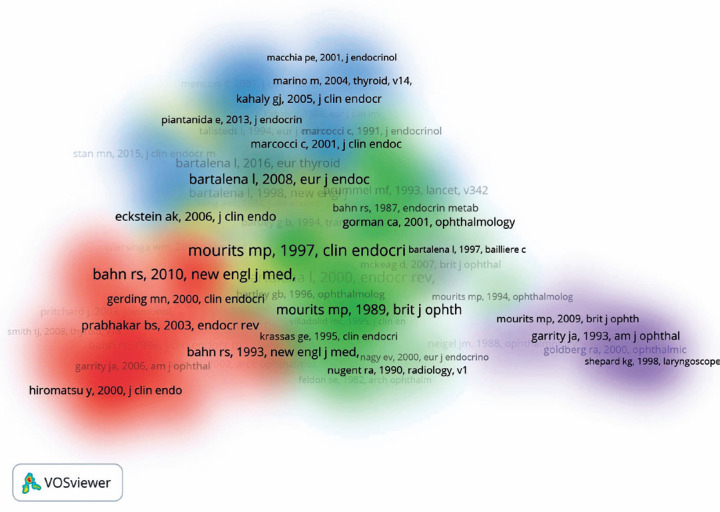
Density visualization map of the co-cited references in TED. Five clusters of co-cited references in different colors.

The 10 highest-cited articles are shown in table 3, with the name of the first author, journal title, citation count, and average citation per year. The article “2016 European Thyroid Association/European Group on Graves’ Orbitopathy Guidelines for the Management of Graves’ Orbitopathy”^(7)^ had the highest number of citations (266) and average citations per year (53.2). Of the 13,211 cited references from TED articles, 233 that were cited at least 20 times were used to create a density visualization map of co-cited references. Five clusters of co-cited references showed up in our analysis: red cluster, references related to pathogenesis; green cluster, references related to TED management; blue cluster, references related to clinical assessment; yellow cluster, references related to risk factors; and purple cluster, references related to the surgical management of TED. The density visualization map showing these five clusters is shown in figure 5.

**Table 3:** The 10 top-cited articles on TED

Title	First author	Journal title	Publication year	Total citations	Average per year
The 2016 European Thyroid Association/European Group on Graves’ Orbitopathy Guidelines for the Management of Graves’ Orbitopathy ^^6^^	Bartalena, L	European Thyroid Journal	2016	266	53.2
Selenium and the Course of Mild Graves’ Orbitopathy	Marcocci, C	New England Journal Of Medicine	2011	250	25
Thyrotropin receptor autoantibodies are independent risk factors for graves’ ophthalmopathy and help to predict severity and outcome of the disease	Eckstein, A	Journal Of Clinical Endocrinology & Metabolism	2006	238	15.87
Epidemiology and prevention of Graves’ ophthalmopathy	Wiersinga, WM	Thyroid	2002	217	11.42
Consensus statement of the European Group on Graves’ Orbitopathy (EUGOGO) on management of Graves’ Orbitopathy	Bartalena, L	Thyroid	2008	215	16.54
Randomized, single blind trial of intravenous versus oral steroid monotherapy in graves’ orbitopathy	Kahaly, GJ	Journal Of Clinical Endocrinology & Metabolism	2005	200	12.5
Association of thyrotrophin receptor antibodies with the clinical features of Graves’ ophthalmopathy	Gerding, MN	Clinical Endocrinology	2000	192	9.14
Comparison of the effectiveness and tolerability of intravenous or oral glucocorticoids associated with orbital radiotherapy in the management of severe Graves’ ophthalmopathy: Results of a prospective, single-blind, randomized study	Marcocci, C	Journal Of Clinical Endocrinology & Metabolism	2001	172	8.6
Radiotherapy for Graves’ orbitopathy: randomised placebo-controlled study	Mourits, MP	Lancet	2000	160	7.62
Graves’ Ophthalmopathy	Bartalena, L	New England Journal Of Medicine	2009	156	13

TED= thyroid eye disease.

TED= thyroid eye disease.

## DISCUSSION

Scientific articles on TED have increased in the past two decades. A strong collaboration between European countries is evident, as they form a cluster on the visualization map; most of the links of any European country are with other European countries. In contrast, the United States collaborates with countries from different clusters, indicating that it has a wider global collaboration in TED research. The two most cited articles in this study were written by a group (European Group of Graves Orbitopathy [EUGOGO]) of authors from different European countries. This finding is in agreement with several studies that found a positive impact of international co-authorship on citation count^(8-10)^.

The country with the highest count of articles and citations is the United States. It is interesting to realize that 10 authors participated in one-third of all TED articles and that 8 of the top 10 authors were from European countries. Wiersinga was the leading author on TED topics in terms of the citation count.

Our analysis determined the leading institutes publishing on TED. This may help scientists who are interested to research this topic to find an appropriate institute for the research. The geographical distributions of these institutes include Italy, the Netherlands, South Korea, the United States, England, and Wales (Table 1).

Bibliometric analysis can help identify the leading journals publishing on TED. It can help scholars publish their articles in journals specializing in this topic. The leading journals in terms of article count are *Thyroid, Ophthalmic Plastic and Reconstructive Surgery*, and the *Journal of Clinical Endocrinology Metabolism*. The leading journals in terms of citation count are the *Journal of Clinical Endocrinology Metabolism, Thyroid*, and *Clinical Endocrinology*. Except for *Ophthalmic Plastic and Reconstructive Surgery*, the top three journals specialize in endocrinology rather than ophthalmology.

Keyword analysis gives good insight into hot research topics. Keyword analysis shows five clusters of keywords that have a common relationship, using which we can identify the common categories of interest in TED research. The first cluster includes keywords related to the pathogenesis of TED. The exact underlying molecular mechanism of TED is unknown, and exploration is in progress. TED is primarily an autoimmune disease^(11)^, and orbital fibroblasts, T-cells, and cytokines, such as thyrotropin receptor and insulin-like growth factor 1 receptor (IGF-1R), play a role in its pathogenesis^(12,13)^. Contrary to fibroblasts in other tissues, orbital fibroblasts express higher levels of CD90 cell surface markers (Thyr-1). Variability in the expression of this marker is also observed within orbital tissue, with higher expression in extraocular muscles compared with adipocytes. Thus, variability results in different cascades of inflammatory reactions^(14)^. Pro-adipocyte subgroups (Thyr-1-negative orbital fibroblasts) express high levels of the thyrotropin receptor. When degraded and presented by antigenpresenting cells, interactions occur between B-cell CD40 and T-cell CD154, resulting in plasma cell production of thyrotropin receptor antibodies. With the conjunction of peroxisome proliferator-activated receptor gamma (PPAR-γ) and interleukin (IL)-6 molecules, thyrotropin receptor antibodies stimulate the maturation of proadipocytes and IL-1 production, while type 1 helper T-cells and IL-1 stimulate Thyr-1-positive fibroblasts to produce hyaluronan and prostaglandins, resulting in extraocular muscle enlargement. In contrast, transforming growth factor beta (TGF-β) stimulates the differentiation of Thyr-1-positive fibroblasts into myofibroblasts and induces fibrosis^(15)^. IGF-1R is overexpressed in Thyr-1positive fibroblasts and plays a role in this inflammation by signaling pathways for IL-16 and regulated upon activation, normal t cell expressed and presumably secreted (RANTES) production. Understanding the underlying disease mechanism is important because will help find drugs that can target the disease. One example is teprotumumab, a novel monoclonal antibody that inhibits IGF-1R. It has been recently approved for the treatment of TED patients^(16,17)^.

The second cluster, in blue, is related to TED management, which includes medical treatment using systemic steroids, radiotherapy and immunosuppressive agents, and surgical treatment^(18)^. In this category, we also encountered keywords of disease activity and the clinical activity score. According to the clinical activity score, we can select an appropriate treatment for each patient^(19)^.

The third cluster is related to surgery. The most used keywords are Graves’ ophthalmopathy, orbital decompression, exophthalmos, proptosis, surgery, diplopia, muscle, and optic neuropathy. The fourth cluster includes keywords related to association, and the main keywords are risk factors, smoking, ocular surface, dry eye, tear film osmolarity, children, glaucoma, intraocular pressure, Graves’ disease, auto-antibodies, thyrotropin receptor antibody, prevalence, and population. We can find the main risk factors for TED in this keyword cluster, including smoking, radioiodine therapy, and hyperthyroidism^(20)^.

Smoking, Graves’ disease duration, female gender, and age of disease onset are important risk factors for TED^(3,21)^. While one study showed that Europeans are at greater risk than Asians to develop TED^(22)^, several other studies did not^(23)^. EUGOGO recommends controlling risk factors as part of TED management, especially smoking and dysthyroid status^(7)^.

The exact molecular mechanism underlying ocular surface abnormalities in TED patients is unknown. The tear film regulatory composition of proteins and cytokines in TED patients is different than in other patients with ocular surface disease; therefore, a different mechanism is proposed^(24)^. As ocular surface disease may be the earliest feature in TED, tear film biomarkers may aid in the early diagnosis of TED^(25)^.

The fifth cluster is related to the quality of life, and the GO-QOL questionnaire is used to assess the quality of life of TED patients^(26)^. TED affects the quality of life negatively due to several complications, such as disfiguring, strabismus, and diplopia, which may cause major depression in the patients. This issue needs to be addressed in TED management^(27-29)^.

Analysis of the keyword average publication year shows the change in research trends. The average publication year of keywords related to the mechanism and associations of TED is 2014, while that of keywords related to the classic model of treatment, such as corticosteroids, and radiotherapy is 2009. The increase in research interest in immunotherapy, such as rituximab, the role of oxidative stress, orbital fibroblasts, proliferation, European group recommendations, and risk factors, is evident in recent years. In contrast, there is less interest in research on radiotherapy and steroid therapy. This may be due to the increased understanding of the molecular mechanism underlying TED, which encourages exploring new treatment targets^(4)^. There is increasing interest in exploring the role of orbital fibroblasts, oxidative stress, nuclear factor kappa B (NF-κB), proliferation, and inflammation in the molecular mechanism underlying TED. The most commonly used synonym for TED is Graves’ ophthalmopathy, which in this study occurred 388 times. The synonym Graves’ orbitopathy has seen a higher average use in recent years. Dysthyroid orbitopathy and endocrine orbitopathy are the least common synonyms and are less commonly used.

Co-citation analysis is an important tool in bibliometric analysis to identify articles that are commonly cited together by several authors. Highly co-cited articles indicate a common relationship. Co-citation analysis also aids in identifying the authors’ input on a particular topic^(30,31)^. In this study, we investigated the topics of highly co-cited articles and found five topics: (1) pathogenesis, (2) management, (3) risk factors, (4) clinical assessment, and (5) surgical management.

Several bibliometric analyses have been carried out to examine research trends in several ophthalmology fields^(32)^. However, to the best of our knowledge, this is the first study to assess research trends in Graves’ orbitopathy.

This study has some limitations, First, we used a single database (WOS). Because the WOS database allows scholars to perform a full-grown analysis of citations, many bibliometric analyses in ophthalmology rely on this database. Second, we limited our analysis to articles published between 2000 and 2019; therefore, older articles were not included. Trends in TED research are continuously changing. Future studies may examine changes in these trends and hot research topics in TED.

## References

[1] Putta-Manohar S, Perros P. (2010). Epidemiology of Graves’ orbitopathy. Pediatr Endocrinol Rev.

[2] Wiersinga WM, Kahaly GJ (2010). Graves’ orbitopathy: A multidisciplinary approach - questions and
answers.

[3] Khong JJ, Finch S, De Silva C, Rylander S, Craig JE, Selva D (2016). Risk Factors for Graves’ Orbitopathy; the Australian Thyroid-
Associated Orbitopathy Research (ATOR) Study. J Clin Endocrinol Metab.

[4] Wu T, Tang DR, Zhao L, Sun FY. (2018). Poly (ADP-ribose) polymerase-1 (PARP-1) in Chinese patients with
Graves’ disease and Graves’ ophthalmopathy. Can J Physiol Pharmacol.

[5] Li Z, Cestari DM, Fortin E. (2018). Thyroid eye disease: what is new to know?. Curr Opin Ophthalmol.

[6] Vavryčuk V. (2018). Fair ranking of researchers and research teams. PLoS One.

[7] Bartalena L, Baldeschi L, Boboridis K, Eckstein A, Kahaly GJ, Marcocci C, European Group on Graves’ Orbitopathy (EUGOGO) (2016). The 2016 European Thyroid Association/European Group on Graves’
Orbitopathy Guidelines for the Management of Graves’
Orbitopathy. Eur Thyroid J.

[8] Glänzel W. (2004). National characteristics in international scientific coauthorship
relations. Scientometrics.

[9] Gazni A, Sugimoto CR, Didegah F. (2012). Mapping world scientific collaboration: Authors, institutions,
and countries. J Am Soc Inf Sci Technol.

[10] Katz J, Hicks D. (2006). How much is a collaboration worth? A calibrated bibliometric
model. Scientometrics.

[11] Douglas RS. (2019). Teprotumumab, an insulin-like growth factor-1 receptor antagonist
antibody, in the treatment of active thyroid eye disease: a focus on
proptosis. Eye (Lond).

[12] Huang Y, Fang S, Li D, Zhou H, Li B, Fan X. (2019). The involvement of T cell pathogenesis in thyroid-associated
ophthalmopathy. Eye (Lond).

[13] Koumas L, Smith TJ, Phipps RP. (2002). Fibroblast subsets in the human orbit: Thy-1+ and Thy-1-
subpopulations exhibit distinct phenotypes. Eur J Immunol.

[14] Xia N, Zhou S, Liang Y, Xiao C, Shen H, Pan H (2006). CD4+ T cells and the Th1/Th2 imbalance are implicated in the
pathogenesis of Graves’ ophthalmopathy. Int J Mol Med.

[15] Bahn RS. (2010). Graves’ ophthalmopathy. N Engl J Med.

[16] Markham A. (2020). Teprotumumab: first Approval. Drugs.

[17] Douglas RS, Kahaly GJ, Patel A, Sile S, Thompson EH, Perdok R (2020). Teprotumumab for the Treatment of Active Thyroid Eye
Disease. N Engl J Med.

[18] Verity DH, Rose GE. (2013). Acute thyroid eye disease (TED): principles of medical and
surgical management. Eye (Lond).

[19] Mourits MP, Prummel MF, Wiersinga WM, Koornneef L. (1997). Clinical activity score as a guide in the management of patients
with Graves’ ophthalmopathy. Clin Endocrinol (Oxf).

[20] Stan MN, Bahn RS. (2010). Risk factors for development or deterioration of Graves’
ophthalmopathy. Thyroid.

[21] McAlinden C. (2014). An overview of thyroid eye disease. Eye Vis (Lond).

[22] Tellez M, Cooper J, Edmonds C. (1992). Graves’ ophthalmopathy in relation to cigarette smoking and
ethnic origin. Clin Endocrinol (Oxf).

[23] Lazarus JH. (2012). Epidemiology of Graves’ orbitopathy (GO) and relationship with
thyroid disease. Best Pract Res Clin Endocrinol Metab.

[24] Novaes P, Diniz Grisolia AB, Smith TJ. (2016). Update on thyroid-associated Ophthalmopathy with a special
emphasis on the ocular surface. Clin Diabetes Endocrinol.

[25] Versura P, Campos EC. (2010). The ocular surface in thyroid diseases. Curr Opin Allergy Clin Immunol.

[26] Terwee CB, Gerding MN, Dekker FW, Prummel MF, Wiersinga WM. (1998). Development of a disease specific quality of life questionnaire
for patients with Graves’ ophthalmopathy: the GO-QOL. Br J Ophthalmol.

[27] Lee H, Roh HS, Yoon JS, Lee SY. (2010). Assessment of quality of life and depression in Korean patients
with Graves’ ophthalmopathy. Korean J Ophthalmol.

[28] Bruscolini A, Sacchetti M, La Cava M, Nebbioso M, Iannitelli A, Quartini A (2018). Quality of life and neuropsychiatric disorders in patients with
Graves’ Orbitopathy: current concepts. Autoimmun Rev.

[29] Farid M, Roch-Levecq AC, Levi L, Brody BL, Granet DB, Kikkawa DO. (2005). Psychological disturbance in graves
ophthalmopathy. Arch Ophthalmol.

[30] Trujillo CM, Long TM. (2018). Document co-citation analysis to enhance transdisciplinary
research. Sci Adv.

[31] Small HG. (1978). Cited Documents as Concept Symbols. Soc Stud Sci.

[32] Boudry C, Baudouin C, Mouriaux F. (2018). International publication trends in dry eye disease research: A
bibliometric analysis. Ocul Surf.

